# Assessing the Effect of Leptin on Liver Damage in Case of Hepatic Injury Associated with Paracetamol Poisoning

**DOI:** 10.1155/2015/357360

**Published:** 2015-11-30

**Authors:** Murat Polat, Serkan Cerrah, Bulent Albayrak, Serkan Ipek, Mahmut Arabul, Fatih Aslan, Omer Yilmaz

**Affiliations:** ^1^Department of Internal Medicine, Erzurum Ataturk University, Yakutiye, 25240 Erzurum, Turkey; ^2^Department of Gastroenterology, Erzurum Ataturk University, Yakutiye, 25240 Erzurum, Turkey; ^3^Department of Gastroenterology, Atatürk Training and Research Hospital, İzmir Katip Çelebi University, Karabaglar, 35360 İzmir, Turkey

## Abstract

*Background Aim*. In case of high-dose acetaminophen intake, the active metabolite can not bind to the glutathione, thereby inducing cellular necrosis through binding to the cytosol proteins. This trial was performed to histologically and biochemically investigate whether leptin was protective against liver damage induced by paracetamol at toxic doses. *Material and Method*. In our trial, 30 female rats, divided into 5 groups, were used. IP leptin administration was performed after an hour in the group of rats, in which paracetamol poisoning was induced. The groups were as follows: Group 1: the control group, Group 2: 20 *µ*g/kg leptin, Group 3: 2 g/kg paracetamol, Group 4: 2 g/kg paracetamol + 10 *µ*g/kg leptin, and Group 5: 2 g/kg paracetamol + 20 *µ*g/kg leptin. *Results*. The most significant increase was observed in the PARA 2 g/kg group, while the best improvement among the treatment groups occurred in the PARA 2 g/kg + LEP 10 *µ*g/kg group (*p* < 0.05). While the most significant glutathione (GSH) reduction was observed in the PARA 2 g/kg group, the best improvement was in the PARA 2 g/kg + LEP 10 *µ*g/kg group (*p* < 0.05). *Conclusion*. Liver damage occurring upon paracetamol poisoning manifests with hepatocyte breakdown occurring as a result of inflammation and oxidative stress. Leptin can prevent this damage thanks to its antioxidant and anti-inflammatory efficacy.

## 1. Introduction

It is known for a long time that high doses of acetaminophen lead to hepatic and renal damage and event death. While the exact mechanism of hepatorenal damage remains unknown, oxidative stress is mentioned most commonly. Acetaminophen is metabolized to its toxic reactive metabolite, N-acetyl-p-benzoquinoneimine (NAPQI). In case of high-dose acetaminophen intake, the active metabolite cannot bind to the glutathione, thereby inducing cellular necrosis through binding to the cytosol proteins [1].

Leptin is the 167-aminoacid hormonal protein product of the obesity gene, which has been investigated thoroughly after being discovered [2]. Leptin, initially described as being associated with being fuel and the energy balance, was suggested to be an antiobesity factor with a feedback effect on the hypothalamus following release from the adipocytes. On the other hand, the study results show that leptin is involved in various physiologic events such as the regulation of metabolism, sexual development, and reproduction [3]. Leptin is known to have a significant role in innate and acquired immunity. The increase of the leptin levels during infection/inflammation suggests that it is a significant factor involved in the host's response to inflammation. Anorexia, observed during the course of the infections, is believed to be the acute phase response of the host. Bacteria/virus products also stimulate the production of the proinflammatory cytokines (interleukins, TNF-alpha, and interferons). Cytokines increase the leptin expression in the fatty tissue. Both microbic products and the resulting cytokines and leptin reduce food intake. Therefore, particularly, TNF-alpha, interleukin-1 (IL-1), and IL-6 are considered to be responsible for inflammation and anorexia occurring during infection and leptin is considered to partially mediate these effects of cytokines [4]. Apart from its various metabolic effects, studies suggest that leptin may also be involved in the regulation of the oxidant/antioxidant balance. Some in vivo and in vitro trials have shown that leptin deficiency was associated with the deficiency of antioxidants. Furthermore, there are findings demonstrating that systemic leptin administration could increase the antioxidant efficacy, deemed to be inadequate in the plasma of people with leptin gene mutation and ob/ob mice with leptin deficiency [5]. Growing evidence underlines the significance of leptin, a hormone that is very important in the regulation of the body weight and food intake both in animals and humans [6]. This trial was performed to histologically and biochemically investigate whether leptin was protective against liver damage induced by paracetamol at toxic doses.

## 2. Material and Method

In our trial, 30 female rats, divided into 5 groups, were used. IP leptin administration was performed after an hour in the group of rats, in which paracetamol poisoning was induced. The groups were as follows: Group 1: the control group, Group 2: 20 *µ*g/kg leptin, Group 3: 2 g/kg paracetamol, Group 4: 2 g/kg paracetamol + 10 *µ*g/kg leptin, and Group 5: 2 g/kg paracetamol + 20 *µ*g/kg leptin. The trial was terminated 24 hours after paracetamol administration and biochemical and histopathological investigations were performed on the tissue and blood samples obtained from the animals.

### 2.1. Experimental Animals

30 Albino Wistar rats, weighing between 195 and 205 grams, provided from the experimental animal laboratory of the Ataturk University Experimental Research and Application Center, were used in the trial. The rats were fed and watered ad libitum (Feed Institution Standard Rat Feed). The animals were kept and fed at room temperature (22°C) in groups at the laboratory prior to the trial. The study's compliance with the ethical rules was approved by the “Ataturk University Animal Experiments Local Ethics Committee.”

### 2.2. Pharmaceuticals and Chemical Substances

Paracetamol (Doğa İlaç Hammaddeleri Ticaret Ltd. Şti.) was used in the study after 2 grams was dissolved in 1% CMC (carboxymethyl cellulose) in PBS (phosphate buffer solution) and mixed under slight heat. It was administered to the rats via IP route.

Leptin, L5037-1 mg (Sigma Aldrich) recombinant, expressed in* E. coli*, was lyophilized; 10 cc was dissolved with 1x PBS. 10 *µ*g/kg and 20 *µ*g/kg doses were administered to the rats via IP route.

Thiopental sodium (IE Ulagay), 50 mg/kg, was administered via IP route for euthanasia.

### 2.3. Analyses of the Liver Tissue

Following the macroscopic analyses, the rat tissues were stored at −80°C. 100 mg tissue from each rat was homogenized in specific homogenate buffer (in appropriate buffer) on ice using Ultra-Turrax and subsequently centrifuged according to the relevant directives. For the biochemical studies, the malondialdehyde (MDA) levels and the glutathione (GSH) levels from each supernatant were measured, respectively, using the high-sensitivity Cell Biolabs OxiSelect TBARS Assay Kit (MDA Quantitation) STA-330 and Cell Biolabs OxiSelect Total Glutathione (GSSG/GSH) Assay Kit STA-312 ELISA kits, specifically designed for the rat tissue in duplicate for each rat liver. In addition, all data were expressed as mean ± standard deviation per mg of protein in all liver supernatants, homogenized with appropriate buffer.

#### 2.3.1. Protein Assay

The protein concentrations were determined via the Lowry method using the commercial protein standards (Sigma Aldrich, Total protein kit-TP0300-1KT, USA).

#### 2.3.2. Serum Analyses

TNF-alpha measurement: blood transferred into the EDTA-containing biochemical tubes was centrifuged at 4000 rpm for 10 minutes at +4°C. The samples were stored at −80°C until the analysis. The TNF-alpha levels of each sample were measured using a high-sensitivity ELISA kit in duplicate (Invitrogen-KRC3011-USA).

### 2.4. AST and ALT Measurement

Blood transferred into the EDTA-containing biochemical tubes was centrifuged at 4000 rpm for 10 minutes at +4°C. The samples were stored at −86°C until the analysis. The AST and ALT levels of each sample were measured using a high-sensitivity ELISA kit in duplicate (USCN Life Science-E90207Ra, E91214Ra (China)).

### 2.5. Histological Analysis

The liver tissues obtained from the rats in all groups were coded and placed in 4% formaldehyde-containing bottles. They were kept in alcohol series at increasing concentrations, then cleaned with xylene, and subsequently kept in paraffin beads in the oven. Then the tissues were embedded in paraffin blocks and prepared for the sectioning procedure. 4-*µ*m sections cut from the paraffin blocks using a microtome (Leica Dsc, Germany) were transferred onto the slides and stained with hematoxylin and eosin (Merck©, Germany). Sections, made ready for investigation, were investigated under a light microscope with an Olympus BH 40 camera attached and photos taken for all relevant groups.

### 2.6. Statistical Analysis

The statistical assessments of the histological studies were performed using the SPSS 18.0 software. The numeric intensity of the necrotic cells was calculated. The data were expressed as mean ± standard deviation. The one-way variance analysis (ANOVA) test and the “Tukey test” were used for the matched experimental groups in the statistical analysis. Values below *p* < 0.05 were considered to be significant.

The statistical analysis of the biochemical studies was conducted using the SPSS 18.0 software. The parametric data were analyzed via one-way variance analysis (ANOVA) test and the “Duncan” technique, a post hoc test. The values obtained were expressed as mean ± standard deviation and *p* values below 0.05 were considered to be statistically significant.

## 3. Results

### 3.1. Histological Results

**Figure 1:**
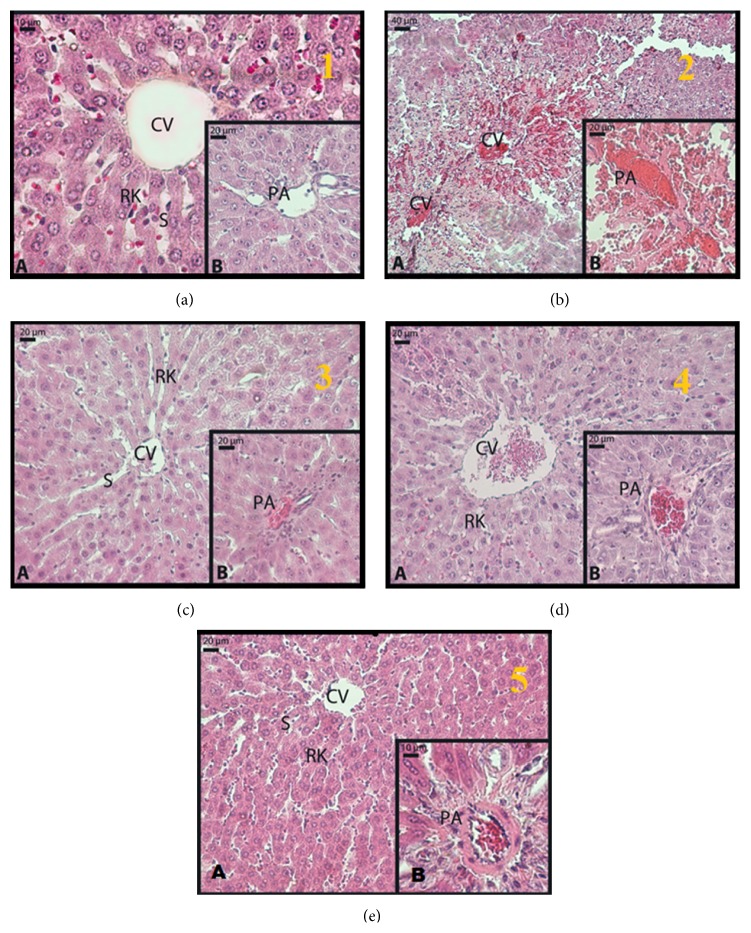
The light microscope images of specimens obtained from the liver samples. (a) Group 1: control group; (b) Group 3: 2 g/kg paracetamol administered group; (c) Group 4: 2 g/kg paracetamol + 10 *µ*g/kg leptin administered group; (d) Group 5: 2 g/kg paracetamol + 20 *µ*g/kg leptin administered group; (e) Group 2: 20 *µ*g/kg leptin administered group (hematoxylin and eosin) (CV: vena centralis, RK: Remark cord, S: sinusoid, PA: portal area).

Investigating the hepatocytes in the parenchyma forming the liver lobules of the experimental animals, the structure was observed to be normal in the control group; that is, these cell cordons, lined up in a radier fashion from the vena centralis to the peripheral lobules (Remark plaques), were recorded as data indicating a normal structure (Figure 1(a), A). Observing the sinusoids, located among the Remark cordons, no structural deformation was detected (Figure 1(a), A and B). The portal regions revealed no abnormality of the vascular structures (Figure 1(a), B).

There were intense necrotic foci in the liver tissue of the animals, which were given paracetamol (Figure 1(b), A). An intense increase of eosinophils in a large number of hepatocytes in the parenchyma, severe hyperchromasia in the nucleus, and irregularity of the cell membrane were observed. Irregular Remark cordons, dilated sinusoids, and intense erythrocyte aggregation were remarkable findings (Figure 1(b), A). In addition, the presence of congestion in both the central vein and the portal area was detected (Figure 1(b), A and B).

Examining the livers of the treated experimental animals, leptin was observed to significantly prevent paracetamol toxicity significantly in the liver. In both 10 *µ*g/kg leptin and 20 *µ*g/kg leptin group, the lining of the hepatocyte cordons from the vena centralis in the parenchyma was quite regular and almost similar to that in the control group (Figure 1(d), A and B). In addition, while the vascular degenerations and congestions occurring in the group with induced paracetamol toxicity almost completely recovered (Figure 1(c), A and B), they were slightly more distinct in the 20 *µ*g/kg leptin group relative to the 10 *µ*g/kg leptin group (Figure 1(d), A and B). However, this distinction was not at a level exceeding the physiologic limits.

The other remarkable regularity in the leptin groups was observed in the control group that was administered 20 *µ*g/kg leptin. Data of this group was in line with that from the control group and the group which was administered 20 *µ*g/kg leptin following paracetamol toxicity (Figure 1(e), A and B).

Making a brief comparison between the doses, we can say that the liver structures were more regular in the 10 *µ*g/kg leptin group compared to the 20 *µ*g/kg leptin group.

**Figure 2:**
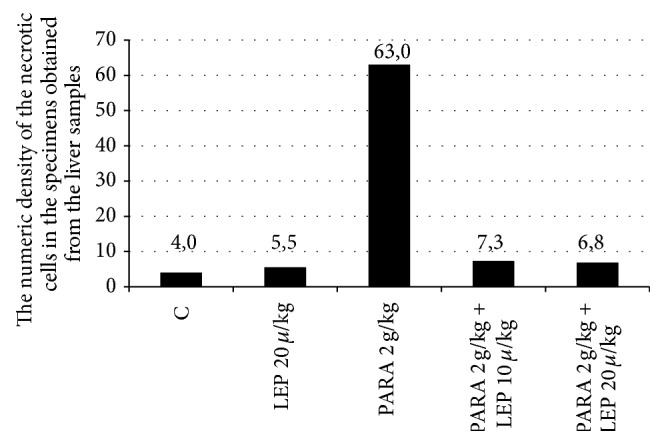
The numeric density of the necrotic cells in the specimens obtained from the liver samples. Group 1: the control group; Group 2: 20 *µ*g/kg leptin; Group 3: 2 g/kg paracetamol; Group 4: 2 g/kg paracetamol + 10 *µ*g/kg leptin; Group 5: 2 g/kg paracetamol + 20 *µ*g/kg leptin. C: control group, LEP: the group receiving leptin, PARA: the group receiving paracetamol. The results were analyzed using the Tukey's technique in the one-way ANOVA test. *p* < 0.05 was considered significant.

As can be seen in Table 1 and Figure 2, in comparison to the control group, the number of the necrotic cells was significantly increased only in the paracetamol group (*p* ≤ 0.001). The number of the necrotic cells identified in the liver tissues of the groups treated with 10 *µ*g/kg and 20 *µ*g/kg leptin was observed to be significantly decreased compared to the paracetamol group (*p* ≤ 0.01). We found a similarity between the leptin 10 *µ*g/kg (*p* = 0.663) and leptin 20 *µ*g/kg (*p* = 0.780) groups and the control group in the number of necrotic cell numbers. The control leptin 20 *µ*g/kg group (*p* = 0.973) was also similar to the control group.

### 3.2. Biochemical Results

#### 3.2.1. AST, ALT and TNF-*α* Measurements

**Figure 3:**
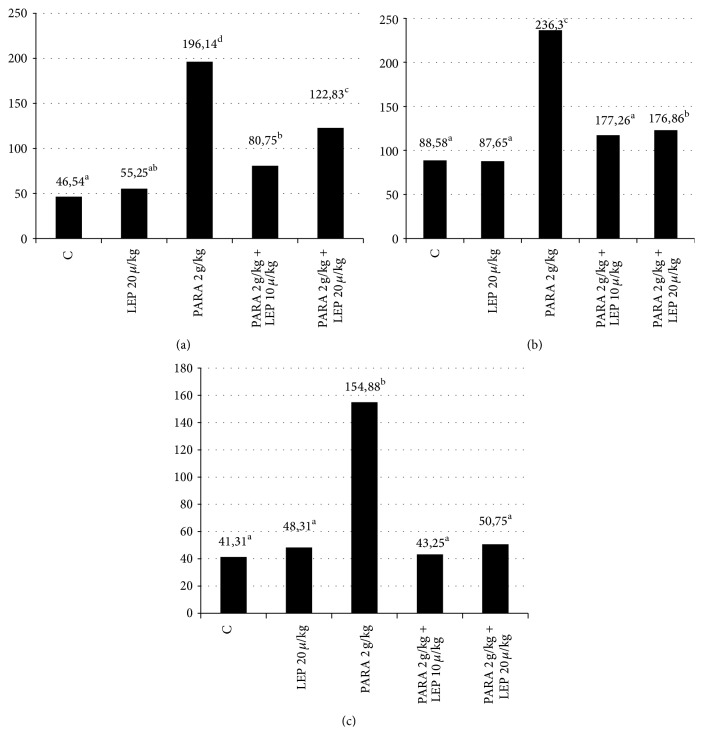
Graphical demonstration of the rat serum ALT level (a); graphical demonstration of the AST level (b); graphical demonstration of the TNF-alpha level (c). Group 1: the control group; Group 2: 20 *µ*g/kg leptin; Group 3: 2 g/kg paracetamol; Group 4: 2 g/kg paracetamol + 10 *µ*g/kg leptin; Group 5: 2 g/kg paracetamol + 20 *µ*g/kg leptin. LEP: the group receiving leptin; PARA: the group receiving paracetamol. There was no statistically significant difference between the values shown with the same letter or letters based on the Duncan multicomparison test. ^a^ *p* < 0.05 comparison of the control group and paracetamol group, ^b^ *p* < 0.05 comparison of the control group and treatment group, and ^c^ *p* < 0.05 comparison of the paracetamol group and the treatment group.

As can be seen in Table 2 and Figure 3, the ALT, AST, and TNF-alpha values are as follows: control group: 46.54 U/L-88.58 U/L-41.31 pg/mL, LEP 20 *µ*g/kg: 55.25 U/L-87.65 U/L-48.31 pg/mL, PARA 2 g/kg group: 196.14 U/L-236.30 U/L-154.88 pg/mL, PARA 2 g/kg + LEP 10 *µ*g/kg group: 80.75 U/L-117.26 U/L-43.25 pg/mL, and PARA 2 g/kg + LEP 20 *µ*g/kg group: 122.83 U/L-176.86 U/L-50.75 pg/mL.

Reviewing the results, the most significant increase was observed in the PARA 2 g/kg group, while the best improvement among the treatment groups occurred in the PARA 2 g/kg + LEP 10 *µ*g/kg group (*p* < 0.05).

#### 3.2.2. GSH and MDA Measurements

**Figure 4:**
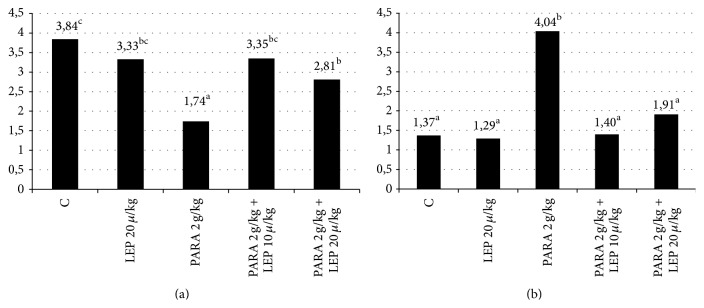
Graphical demonstration of the rat serum GSH level (a); graphical demonstration of the MDA level (b). Group 1: the control group; Group 2: 20 *µ*g/kg leptin; Group 3: 2 g/kg paracetamol; Group 4: 2 g/kg paracetamol + 10 *µ*g/kg leptin; Group 5: 2 g/kg paracetamol + 20 *µ*g/kg leptin. LEP: the group receiving leptin, PARA: the group receiving paracetamol. Malonaldehyde (MDA) levels and the glutathione (GSH). The results were analyzed using the post hoc Duncan technique in the one-way ANOVA test. ^a^ *p* < 0.05 comparison of the control group and paracetamol group, ^b^ *p* < 0.05 comparison of the control group and treatment group, and ^c^ *p* < 0.05 comparison of the paracetamol group and the treatment group. *p* < 0.05 was considered significant.

As can be seen in Table 3 and Figure 4, the GSH and MDA levels measured in the liver tissues obtained from the experimental groups are as follows: control group, 3.84-1.37 nmol/mg; LEP 20 *µ*g/kg group, 3.33-1.29 nmol/mg; PARA 2 g/kg group, 1.74-4.04 nmol/mg; PARA 2 g/kg + LEP 10 *µ*g/kg group, 3.35-1.40 nmol/mg; PARA 2 g/kg + LEP 20 *µ*g/kg group, 2.81-1.91 nmol/mg.

While the most significant GSH reduction was observed in the PARA 2 g/kg group, the best improvement was in the PARA 2 g/kg + LEP 10 *µ*g/kg group (*p* < 0.05). While the MDA level was observed to significantly increase in the PARA 2 g/kg group, the best improvement was in the PARA 2 g/kg + LEP 10 *µ*g/kg group among the treatment groups (*p* < 0.05).

## 4. Discussion

Despite the abundance of trials on paracetamol toxicity, the mechanism of the liver cell damage has not been elucidated yet. Based on the commonly acceptable opinion, this damage process starts upon paracetamol being metabolized to its reactive metabolite, NAPQI. This metabolite first depletes GSH as a result of being increased in the blood followed by binding to various cellular proteins, which also include the mitochondrial proteins. As a result of this process, suppression of the mitochondrial respiration, consumption of the adenosine triphosphate (ATP), and mitochondrial oxidative stress may occur. ATP consumption results in cellular oncotic necrosis in the hepatocytes and the sinusoidal endothelial cells [7].

Aspartate aminotransferase and alanine aminotransferase are the intracellular enzymes of the liver and the most reliable two parameters, which increase in case of hepatocellular damage or necrosis [8]. In our trial, we detected a three- to fourfold increase in the AST and ALT values of the group with induced toxicity in the measurements performed on the blood samples obtained from the experimental animals after 24 hours. These results were in compliance with the results obtained from the other trials [9–12]. The AST and ALT values, measured in the groups receiving leptin, were detected to be markedly low relative to the group with induced toxicity. This finding shows that leptin can be enzymatically protective against the liver damage occurring as a result of paracetamol toxicity.

Lipid peroxidation is one of the most significant mechanisms of paracetamol-associated liver damage and is secondary to the free oxygen radicals as a result of oxidative stress. Lipid peroxidation results from the effect of the free radicals on the multi-unsaturated fatty acids. f is the final product of the lipid peroxidation and is a marker, which is commonly used in showing lipid peroxidation. MDA levels increase in tissues, exposed to oxidative stress. This shows that the plasma MDA level can be used as a biomarker for oxidative stress [13]. The MDA value of the liver tissue was detected to be increased in the group with induced paracetamol toxicity and the values obtained were in line with the results from the recent relevant trials [14–16]. In our trial, we detected that the increased oxidative stress could be prevented by leptin. In our trial, we also observed that the blood MDA levels did not significantly increase in rats that were treated with leptin. In line with the results from other trials, this finding showed that leptin created defense against the paracetamol-associated oxidative stress and prevented lipid peroxidation and thus could be effective in maintaining the integrity of the cell membrane [17–19].

Glutathione is one of the most significant antioxidants, which is involved in cellular protection against oxidative stress. GSH is available in the reduced and oxidized forms. Reduced GSH has the ability to give the unstable molecules, such as the reactive oxygen products, the reducing equivalents. The protective effect occurs via this mechanism. In cases such as reduced cellular GSH levels and GSH synthesis capacity, the cells become susceptible to oxidative stress. At the toxic doses of paracetamol, NAPQI, as a mediator of oxidative stress, caused a reduction in the GSH levels and thus hepatic damage [20]. In our trial, the tissue GSH value was observed to be decreased in case of paracetamol-induced hepatotoxicity [21–23]. In our trial, we detected that the reduction in the GSH values was detected to be statistically significantly avoided in the leptin-treated groups. This finding supports that leptin provides protection against the oxidative stress occurring in the hepatocytes in case of paracetamol poisoning and that it had an antioxidant capacity and reduced cellular damage.

In case of liver necrosis secondary to paracetamol toxicity, serum cytokines and particularly TNF-alpha together with oxidative stress were shown to be involved by the recent trials [24, 25]. TNF-alpha is a proinflammatory cytokine, mostly produced by the macrophages in the liver. It is the primary mediator of systemic toxicity and liver damage. TNF-alpha is known to have a significant role in many forms of liver damage, including ischemia, reperfusion, and fulminant hepatic failure. In addition to initiating the aggressive inflammatory process and deteriorating the cellular damage further, it also acts as a central regulator that stimulates apoptosis and cell proliferation and contributes to tissue repair [26]. In our trial, the TNF-alpha level was observed to be increased 4-fold in the group receiving paracetamol relative to the other groups. This indicates that TNF-alpha significantly increases as a result of the paracetamol toxicity [16, 27, 28]. Our trial results are in compliance with those from the literature trials that show that leptin has an anti-inflammatory efficacy [29, 30] because our leptin groups demonstrated a significant improvement in the TNF-alpha level. This proves that the proinflammatory effects of TNF-alpha, significantly involved in the liver damage, could be prevented by leptin and that exogenous leptin has an anti-inflammatory efficacy.

Liver damage secondary to paracetamol toxicity does not only lead to enzymatic changes but also to histological changes. The use of paracetamol at toxic doses results in the development of centrilobular necrosis [31]. Additionally, at supratherapeutic doses, chronic drug administration was associated with various levels of centrilobular necrosis in the liver and depending on the time and the damage spread to the other regions of the liver lobules [32]. In our trial, intense necrotic foci were observed in line with the other trials performed using paracetamol. Particularly, an intense eosinophil increase in the cytoplasm of a large number of hepatocytes in the parenchyma, severe hyperchromasia in the nucleus, and irregularity of the cell membrane were detected. Irregular Remark cordons, dilated sinusoids, and intense erythrocyte aggregation were remarkable findings. In addition, the presence of congestion in the vascular structures in both the central vein and the portal region was among the other pathological changes observed [11, 12, 21, 33–35]. Reviewing the treatment groups, leptin was observed to significantly prevent paracetamol toxicity in the liver. In both 10 *µ*g/kg leptin and 20 *µ*g/kg leptin groups, the lining of the hepatocyte cordons from the vena centralis in the parenchyma was quite regular and almost similar to that in the control group. Additionally, investigating the hepatocytes closer, no necrotic cells were detected in the group receiving paracetamol; the hepatocytes appeared regular. While the vascular degenerations and congestions occurring in the group with induced paracetamol toxicity almost completely recovered, they were slightly more distinct in the 20 *µ*g/kg leptin group relative to the 10 *µ*g/kg leptin group. However, this distinction was not at a level to exceed the physiologic limits. The similarity of our findings to the results indicating that the substances used in the above trials are protective against paracetamol toxicity shows that leptin has a histologically protective effect against liver damage occurring upon paracetamol poisoning.

In our trial, we administered leptin at the 10 *µ*g/kg and 20 *µ*g/kg doses. The higher level of histological and biochemical improvement observed in the 10 *µ*g/kg leptin group relative to the 20 *µ*g/kg leptin suggests that this dose could be the therapeutic dose of leptin in this indication and that the 20 *µ*g/kg leptin dose could correspond to the supratherapeutic doses.

In conclusion, liver damage occurring upon paracetamol poisoning manifests with hepatocyte breakdown occurring as a result of inflammation and oxidative stress. Leptin can prevent this damage thanks to its antioxidant and anti-inflammatory efficacy. However, further trials are needed to be conducted on this subject.

## Figures and Tables

**Table 1:** The numeric density of the necrotic cells in the specimens obtained from the liver samples.

Group	Numeric density of the necrotic hepatocytes (cm^2^)
Control	4 ± 1.41^a.b^
LEP 20 *µ*g/kg	5.5 ± 1.87^b.c^
PARA 2 g/kg	63 ± 16.26^a.c^
PARA 2 g/kg + LEP 10 *µ*g/kg	7.3 ± 1.21^b.c^
PARA 2 g/kg + LEP 20 *µ*g/kg	6.83 ± 2.48^b.c^

Group 1: the control group; Group 2: 20 *µ*g/kg leptin; Group 3: 2 g/kg paracetamol; Group 4: 2 g/kg paracetamol + 10 *µ*g/kg leptin; Group 5: 2 g/kg paracetamol + 20 *µ*g/kg leptin. LEP: the group receiving leptin, PARA: the group receiving paracetamol. ^a^ *p* < 0.05 comparison of the control group and paracetamol group, ^b^ *p* < 0.05 comparison of the control group and treatment group, and ^c^ *p* < 0.05 comparison of the paracetamol group and the treatment group. *p* < 0.05 was considered significant.

**Table 2:** ALT and AST levels measured in the rat sera.

Group	ALT (U/L)	AST (U/L)	TNF-*α* (pg/mL)
Control	46.54 ± 11.67^a^	88.58 ± 21.03^a^	41.31 ± 2.36^a^
LEP 20 *µ*g/kg	55.25 ± 13.99^a.b^	87.65 ± 11.08^a^	48.31 ± 9.67^a^
PARA 2 g/kg	196.14 ± 53.80^d^	236.30 ± 37.18^c^	154.88 ± 34.14^b^
PARA 2 g/kg + LEP 10 *µ*g/kg	80.75 ± 11.67^b^	117.26 ± 25.31^a^	43.25 ± 9.53^a^
PARA 2 g/kg + LEP 20 *µ*g/kg	122.83 ± 39.81^c^	176.86 ± 57.44^b^	50.75 ± 12.25^a^

Group 1: the control group, Group 2: 20 *µ*g/kg leptin. Group 3: 2 g/kg paracetamol. Group 4: 2 g/kg paracetamol + 10 *µ*g/kg leptin. Group 5: 2 g/kg paracetamol + 20 *µ*g/kg leptin. LEP: the group receiving leptin, PARA: the group receiving paracetamol. The results were analyzed using the post-hoc Duncan technique in the one-way ANOVA test. ^a^ *p* < 0.05 comparison of the control group and paracetamol group, ^b^ *p* < 0.05 comparison of the control group and treatment group, ^c^ *p* < 0.05 comparison of the paracetamol group and the treatment group (PARA 2 g/kg + LEP 10 *µ*g/kg), ^d^ *p* < 0.05 comparison of the paracetamol group and the treatment group (PARA 2 g/kg + LEP 20 *µ*g/kg), *p* < 0.05 was considered significant.

**Table 3:** The GSH and MDA levels measured in the rat liver tissues.

Group	GSH (nmol/mg protein)	MDA (nmol/mg protein)

Control	3.84 ± 0.79^c^	1.37 ± 0.23^a^
LEP 20 *µ*g/kg	3.33 ± 0.72^b.c^	1.29 ± 0.76^a^
PARA 2 g/kg	1.74 ± 0.55^a^	4.04 ± 1.50^b^
PARA 2 g/kg + LEP 10 *µ*g/kg	3.35 ± 0.71^b.c^	1.40 ± 0.78^a^
PARA 2 g/kg + LEP 20 *µ*g/kg	2.81 ± 0.66^b^	1.91 ± 0.40^a^

Group 1: the control group; Group 2: 20 *µ*g/kg leptin; Group 3: 2 g/kg paracetamol; Group 4: 2 g/kg paracetamol + 10 *µ*g/kg leptin; Group 5: 2 g/kg paracetamol + 20 *µ*g/kg leptin. Malondialdehyde (MDA) levels and the Glutathione (GSH). LEP: the group receiving leptin, PARA: the group receiving paracetamol. The results were analyzed using the post hoc Duncan technique in the one-way ANOVA test. ^a^ *p* < 0.05 comparison of the control group and paracetamol group, ^b^ *p* < 0.05 comparison of the control group and treatment group, and ^c^ *p* < 0.05 comparison of the paracetamol group and the treatment group. *p* < 0.05 was considered significant.
